# Assessing the Diagnostic Performance of CT in Suspected Urinary Stones: A Retrospective Analysis

**DOI:** 10.7759/cureus.37699

**Published:** 2023-04-17

**Authors:** Mahdi Aljawad, Fatimah A Alaithan, Besma S Bukhamsin, Adnan A Alawami

**Affiliations:** 1 Radiology, Qatif Central Hospital, Qatif, SAU; 2 Radiology, Dammam Medical Complex, Dammam, SAU; 3 Radiology, King Fahd Hospital of the University, Al-Khobar, SAU; 4 Radiology, Royal Commission Hospital, Jubail, SAU

**Keywords:** ureteric stones, renal stones, retrospective study, urologic interventions, positive rate, computed tomography, emergency department, urinary stone disease

## Abstract

Background

Urinary stone disease is a common reason for emergency department (ED) visits, and a computed tomography scan of the kidneys, ureters, and bladder (CT-KUB) is frequently used for diagnosis. The objective of this study was to estimate the positive rate of CT-KUB and identify predictors of emergency interventions for patients with ureteric stones.

Methods

A retrospective study was conducted to investigate the positive rate of CT-KUB for urinary stone disease and to explore the factors that determine the need for emergency urologic interventions. The study population included adult patients who underwent CT-KUB to rule out urinary stones at King Fahd University Hospital.

Results

The study included 364 patients, of whom 245 (67.3%) were men and 119 (32.7%) were women. CT-KUB revealed stones in 243 (66.8%) patients, including 32.4% with renal stones and 54.4% with ureteric stones. Female patients were more likely to have normal results than male patients. Approximately 26.8% of patients with ureteric stones required emergency urologic intervention. Multivariable analysis found that the size and location of ureteric stones were independent predictors for emergency intervention. Patients with distal ureteric stones were 35% less likely to need emergency interventions than those with proximal stones.

Conclusion

The positive rate of CT-KUB was acceptable for patients with suspected urinary stone disease. Most demographic and clinical characteristics were not predictors for emergency interventions, but the size and location of ureteric stones and elevated creatinine levels were significantly associated.

## Introduction

Urinary stone disease is a prevalent and steadily increasing problem worldwide, imposing significant clinical and economic burdens [1]. Renal colic represents a common cause of visits to the emergency department (ED), accounting for over one million ED visits in the United States [2]. The gold standard for diagnosing urinary stone disease is a non-contrast computed tomography scan of the kidneys, ureters, and bladder (CT-KUB), due to its excellent diagnostic performance with a sensitivity exceeding 94% [3]. However, CT-KUB exposes patients to high levels of ionizing radiation and potential cancer risk [4]. The positive rate for urinary stones may be considered an indicator for the appropriate use of CT-KUB, and published studies report that CT-KUB should detect urinary stones in 44-64% of patients [5,6].

The prevalence of urinary stone disease has marked variability according to demographic and geographic factors [7]. Previous research has indicated a consistent association between mean ambient temperature and the incidence of urinary stones [8]. For instance, Borg et al. [9] demonstrated that a 1 °C increase in daily temperature was significantly associated with kidney disease, including urinary stone disease. However, no study to date has examined the positive rate of CT-KUB in any institution in Saudi Arabia or the Gulf Region, which has a dry desert climate with very high temperatures throughout the year. Dehydration is a well-known contributor to the formation of urinary stones [10]. Investigating the association of hematocrit, a marker of hydration status, with urinary stone disease is of particular interest.

Therefore, this study aims to estimate the positive rate of CT-KUB for urinary stone disease in an academic institution in Saudi Arabia based on different demographic and clinical factors. Additionally, the study aims to determine the factors that are independently associated with the need for urologic interventions in patients presenting to the ED with suspected urinary stones.

## Materials and methods

Study design and setting

We conducted a retrospective study to investigate the positive rate of CT-KUB for urinary stone disease and explore the determinants of the need for emergency urologic interventions. The study was conducted at the King Fahd Hospital of Imam Abdulrahman Bin Faisal University, an academic center located in the Eastern Province of Saudi Arabia.

Study population

Using the Radiology Information System, we identified all requests for CT-KUB scans for patients presenting at the ED between January 2019 and December 2020. Eligible participants were adult patients who underwent CT-KUB to rule out urinary stones. The exclusion criteria were age below 18 years and the purpose of the scan not being to rule out urinary stone disease.

Data collection

We collected data from electronic health records using a structured data collection form. The form included exposure variables such as demographic, clinical, and radiological information as well as outcome variables such as the presence of ureteric stones on CT-KUB scans and the need for emergency urologic intervention within 48 hours of presentation to the ED.

The demographic data included age, sex, smoking status, body mass index, and previous history of urinary stone disease. Clinical information included the duration of symptoms and vital signs at presentation. Laboratory findings included hematocrit, leukocyte count, urea, creatinine, and urinalysis results. Imaging findings included a plain radiograph, an ultrasound examination, and CT-KUB findings. The CT-KUB scans were reviewed to determine the size and site of urinary stones.

Statistical analysis

We analyzed the data using IBM SPSS for Windows, version 26 (IBM Corp., Armonk, NY, USA). We presented all variables as percentages and frequency distributions and compared them using the chi-squared or Fisher’s exact tests, as appropriate. Laboratory biomarkers were dichotomized according to the upper limit of the reference range. Hematocrit values were dichotomized using receiver operating characteristic analysis for each gender separately. We used a figure to illustrate a summary of the CT-KUB findings. We conducted a multivariable binary logistic regression analysis to identify the independent factors associated with needing emergency urologic intervention. Candidate variables were selected based on risk factors identified in the literature and bivariate analyses. We estimated odds ratios with 95% confidence intervals using the full model fit and reported them in comparison with the designated reference group. The significance level was defined as α = 0.05.

## Results

Patient characteristics

The study included 364 patients (67.3% men, 32.7% women), aged 18 to 50 years, with 25.5% of patients having a history of urinary stones. Flank pain was the most frequent symptom reported (79.4%). Hematuria was present in 11.5% of patients, and 51.6% of patients had pain for more than 24 hours before presentation. 30.2% of patients were admitted, and 14.6% underwent emergency urologic intervention (Table 1).

**Table 1:** Characteristics of patients N: number of patients

Variable		N	(%)
Age (years)	18–35	147	(40.4)
	36–50	110	(30.2)
	51–65	78	(21.4)
	>65	29	(8.0)
Gender	Male	245	(67.3)
	Female	119	(32.7)
History of urinary stones	Yes	93	(25.5)
	No	271	(74.5)
Presenting symptoms	Flank pain	289	(79.4)
	Abdominal pain (non-flank)	58	(15.9)
	Nausea	61	(16.8)
	Vomiting	80	(22.0)
	Hematuria	42	(11.5)
	Dysuria	67	(18.4)
Duration of pain (hours)	<6	70	(24.2)
	6–24	70	(24.2)
	>24	149	(51.6)
Heart rate (bpm)	≤100	308	(85.1)
	>100	54	(14.9)
Temperature (℃)	<38	350	(96.7)
	≥38	12	(3.3)
Emergency intervention	Yes	53	(14.6)
	No	311	(85.4)
Disposition decision	Admission	110	(30.2)
	Discharge	254	(69.8)

Initial investigations

Of the patients, 92.0% underwent urinalysis before CT-KUB, with 55.5% of these patients having positive results for blood. Elevated creatinine levels were observed in 35.9% of patients on presentation. Plain radiographs were performed on 63.7% of patients before CT-KUB, and 31.8% of them had stones. Abdominal ultrasound was performed in only 16.2% of patients, of whom 54.2% showed hydronephrosis (Table 2).

**Table 2:** Imaging and laboratory findings of patients N: number of patients; CT: computed tomography; IQR: interquartile range

Variable				N	(%)
Urinalysis	Not done			29	(8.0)
	Blood			186	(55.5)
	Leukocytes			76	(22.7)
	Nitrites			20	(6.0)
Plain radiograph	Not done			232	(63.7)
	Urinary stone			42	(31.8)
Renal ultrasound	Not done			305	(83.8)
	Renal stone			7	(11.9)
	Hydronephrosis			32	(54.2)
CT findings	Urinary stones			243	(66.8)
		Renal		118	(32.4)
		Ureteric		198	(54.4)
			Proximal	64	(33.2)
			Middle	23	(11.9)
			Distal	106	(54.9)
	Alternative diagnosis			34	(10.2)
	Normal scan			84	(23.1)
Hematocrit (%)	Elevated			75	(21.9)
	Non-elevated			267	(78.1)
Leukocytes count (cells/mL)	Elevated			274	(76.5)
	Non-elevated			84	(23.5)
Blood urea nitrogen (mg/dL)	Elevated			313	(87.2)
	Non-elevated			46	(12.8)
Creatinine (mg/dL)	Elevated			230	(64.1)
	Non-elevated			129	(35.9)

Computed tomography findings

Urinary stones were found in 243 (66.8%) patients, with renal stones in 32.4% and ureteric stones in 54.4% of patients. The remaining CT-KUB scans showed alternative diagnoses in 10.2% of patients and were normal in 23.1%. Gender differences were observed, with 73.9% of male patients having urinary stones compared to 52.1% of female patients. Females were more likely to have normal findings (36.1% vs. 16.7%; P < 0.05).

Factors associated with ureteric stones

Ureteric stones were found in 54.4% of patients, with 54.9% of them being in the distal third of the ureter. Factors significantly associated with ureteric stones included male gender, flank pain, hematuria, blood in urinalysis, elevated hematocrit, and high blood urea nitrogen levels (P < 0.05). One-third of the 198 patients with ureteric stones had a history of urinary stones (P < 0.05) (Table 3).

**Table 3:** Factors associated with ureteric stones N: number of patients; CT: computed tomography

Variable		Ureteric stone on CT		P-value
		N	(%)	
Age (years)	18–35	86	(56.5)	0.005
	36–50	67	(60.9)	
	51–65	41	(52.6)	
	>65	7	(24.1)	
Gender	Male	150	(61.2)	<0.001
	Female	48	(40.3)	
History of urinary stones	Yes	64	(68.8)	0.001
	No	134	(49.4)	
Presenting symptoms	Flank pain	169	(58.5)	0.002
	Abdominal pain (non-flank)	28	(48.3)	0.307
	Nausea	30	(49.2)	0.370
	Vomiting	50	(62.5)	0.099
	Hematuria	14	(33.3)	0.004
	Dysuria	29	(43.3)	0.043
Duration of pain (hours)	<6	27	(38.6)	0.205
	6–24	24	(34.3)	
	>24	69	(46.3)	
Urinalysis	Blood	114	(61.3)	0.013
	Leucocytes	35	(46.1)	0.067
	Nitrites	10	(50.0)	0.628
Plain Radiograph	Stones	37	(41.1)	<0.001
Ultrasound	Stones	6	(85.7)	0.221
	Hydronephrosis	23	(71.9)	0.016
Tachycardia	Heart Rate >100 bpm	19	(35.2)	0.002
Fever	Temperature ≥ 38℃	4	(33.3)	0.131
Hematocrit	Elevated	173	(64.8)	<0.001
	Non-Elevated	20	(26.7)	
Leukocytes Count	Elevated	45	(53.6)	0.759
	Non-Elevated	152	(55.5)	
Blood Urea Nitrogen	Elevated	10	(21.7)	<0.001
	Non-Elevated	188	(60.1)	
Creatinine	Elevated	71	(55.0)	0.974
	Non-Elevated	127	(55.2)	

Emergency urologic interventions for ureteric stones

Overall, 26.8% of patients with ureteric stones required emergency urologic intervention, with patients having ureteric stones greater than 4 mm, distal ureteric stones, and elevated creatinine levels more likely to require intervention (P < 0.05). The rate of intervention success was 89.7%, with only six patients requiring repeat interventions due to residual or recurrent stones. The complications of the intervention were rare, with only one patient experiencing postoperative bleeding requiring a blood transfusion (Table 4).

**Table 4:** Factors associated with emergency intervention in patients with ureteric stones N: number of patients; CT: computed tomography

Variable		Emergency intervention		P-value
		N	(%)	
Age (years)	18–35	12	(14.5)	0.205
	36–50	19	(28.4)	
	51–65	9	(22.0)	
	>65	1	(14.3)	
Gender	Male	34	(22.7)	0.229
	Female	7	(14.6)	
History of urinary stones	Yes	23	(35.9)	<0.001
	No	18	(13.4)	
Presenting symptoms	Flank pain	32	(18.9)	0.137
	Abdominal pain (non-flank)	9	(32.1)	0.107
	Nausea	2	(6.7)	0.039
	Vomiting	8	(16.0)	0.342
	Hematuria	2	(14.3)	0.538
	Dysuria	8	(27.6)	0.322
Duration of pain (hours)	<6	4	(9.3)	0.164
	6–24	11	(23.9)	
	>24	17	(21.3)	
Urinalysis	Blood	22	(19.3)	0.235
	Leucocytes	7	(20.0)	0.732
	Nitrites	1	(10.0)	0.341
Plain radiograph	Stones	6	(17.6)	0.872
Ultrasound	Stones	1	(16.7)	0.559
	Hydronephrosis	3	(13.0)	0.738
Site of ureteric stone	Proximal	19	(29.7)	0.029
	Middle	7	(30.4)	
	Distal	15	(14.2)	
Size of ureteric stone >4 mm		27	(34.6)	<0.001
Tachycardia		2	(10.5)	0.249
Fever		0	(0.0)	0.302
Elevated hematocrit		38	(22.0)	0.471
Elevated leukocytes count		7	(15.6)	0.323
Elevated blood urea nitrogen		3	(30.0)	0.435
Elevated creatinine		23	(32.4)	0.002

Factors associated with emergency interventions

In total, 35 (31.8%) patients who underwent emergency interventions experienced adverse outcomes, including failed interventions, prolonged hospitalization, or postoperative complications. Several factors were found to be significantly associated with adverse outcomes. The size of the stone was found to be significantly associated with adverse outcomes, with patients having stones larger than 10 mm being more likely to experience adverse outcomes (63.6%) compared with those with stones not larger than 4 mm (16.7%; P < 0.05). Additionally, patients with proximal ureteric stones were more likely to experience adverse outcomes (54.5%) compared with those with middle (31.3%) or distal (25.0%) ureteric stones (P = 0.03). Patients with elevated creatinine levels were more likely to experience adverse outcomes (45.5%) compared with those with normal creatinine levels (26.7%; P = 0.05). Furthermore, patients with bilateral ureteric stones were more likely to experience adverse outcomes (66.7%) compared with those with unilateral stones (27.5%; P < 0.05) (Table 5).

**Table 5:** Multivariable analysis of factors associated with emergency intervention in patients with ureteric stones OR: odds ratio; CI: confidence interval

Variable		Multivariable regression analysis		
		OR	95% CI	P-value
Age (years)	18–35	Reference group		
	36–50	1.10	[0.37–3.29]	0.862
	51–65	2.07	[0.62–6.86]	0.237
	>65	4.34	[0.19–98.2]	0.356
Male gender		0.45	[0.14–1.50]	0.186
History of urinary stones		2.13	[0.84–5.41]	0.110
Duration of pain (hours)	<6	Reference group		
	6–24	4.24	[1.00–17.90]	0.050
	>24	2.53	[0.59–8.62]	0.235
Site of ureteric stone	Proximal	Reference group		
	Middle	1.41	[0.39–5.08]	0.602
	Distal	0.35	[0.12–1.00]	0.048
Size of ureteric stone >4 mm		5.68	[2.09–15.43]	0.001
Creatinine >1.3 mg/dL		3.72	[1.41–9.81]	0.008

## Discussion

The study aimed to assess the positivity rate of CT-KUB for urinary stone disease and investigate the independent factors associated with the need for emergency urologic interventions in patients with ureteric stones.

Positivite rate of CT-KUB

The study revealed that 66.8% of CT-KUB scans were positive for urinary stone disease. There was a notable difference in the positivity rate of CT-KUB according to gender, with female patients having a higher number of normal scans. The lower positivity rate of CT-KUB in female patients is a consistent finding in previous research [5,11,12]. Hence, it is often advised that female patients undergo a proper clinical assessment with urinalysis and an initial ultrasound examination [3,13]. While ultrasound examination is safe with no radiation exposure, it has limited utility in detecting small stones with low-grade hydronephrosis [14,15]. Similarly, patients without hematuria can have a urinary stone disease with a higher incidence of obstructive uropathy [16]. It is worth noting that the positivity rate of 52.1% for urinary stones in female patients in our study is considered an acceptable rate in view of prior studies [5,6]. For instance, Yong [17] reported a positivity rate of 18% in female patients who underwent CT-KUB at the Royal Infirmary of Edinburgh. Furthermore, in a retrospective study involving over 1,300 patients who underwent CT-KUB, the positivity rate for urinary stones among female patients was 26.8% [11].

The findings showed that over two-thirds of patients with a positive CT-KUB for urinary stones had a previous history of stone disease. It is estimated that one-third of patients with acute urinary stone disease may develop recurrence despite advances in management [18]. Accordingly, it is recommended that CT-KUB be avoided in young patients with a history of urinary stone disease who present with a clinical picture of uncomplicated stone disease [19]. A subgroup analysis was conducted in our study on patients with a previous history of urinary stone disease, which revealed that most of these patients had no clinical or laboratory findings suggestive of complicated stone disease necessitating the repeat of the CT-KUB. Alternatively, plain radiographs can be used for follow-up [20].

Higher hematocrit values were significantly associated with ureteric stones on CT-KUB. The hematocrit value is an indirect index of hemoconcentration and dehydration [21], which are key contributors to urinary stone formation [10]. A similar conclusion was reached by Fukuhara et al. [22], who demonstrated that the addition of the seasonal variable into their predictive model for urinary stone disease significantly enhanced the diagnostic performance of the model.

Factors associated with emergency interventions

The study revealed that most of the demographic and clinical characteristics of patients were not independently associated with the need for emergency urologic intervention. However, the size of the ureteric stone was an independent factor associated with the need for emergency intervention. This finding ties well with previous studies wherein a small ureteric stone was a strong predictor of spontaneous passage of the stone without the need for urologic interventions [13]. The distal location of the ureteric stone is also an important factor associated with a lower rate of emergency interventions [23].

It is interesting to note that an elevated creatinine level was an independent factor associated with urologic interventions; however, the creatinine level was not associated with the presence of the ureteric stone itself. We speculate that this finding could be related to the low threshold for requesting CT-KUB in the ED for patients with deranged renal function tests. Since acute renal injury is common, reaching up to 10% of patients presenting at the ED [24], the isolated serum creatinine measurement cannot be a reliable indicator for urinary stones.

The duration of pain was among the factors that we expected to have been related to emergency interventions but was not found to be independently associated with our study. This may indicate the weak correlation between the reported pain and the disease severity or complications, considering its subjective nature [25]. In contrast, in a prospective study by Papa et al. [26], they found that the pain score was associated with urologic intervention within four weeks after the initial presentation at the ED.

Strengths and limitations

This study is the first to investigate the positive rate of CT-KUB for urinary stones among patients presenting at the ED in Saudi Arabia and the Gulf region. Furthermore, it was carried out in one of the largest hospitals in the Eastern Province of Saudi Arabia. However, the present study has certain limitations. The single-center and retrospective nature of the study mandates larger prospective studies to validate the findings.

## Conclusions

The study found that the positive rate of CT-KUB was acceptable for both male and female patients presenting with suspected urinary stone disease at the ED of the institution. Patients with ureteric stones had higher hematocrit values compared to those with normal imaging findings. While most demographic and clinical characteristics were not independently associated with the need for emergency urologic interventions, the size and location of the ureteric stones and elevated creatinine levels were found to be significant factors. Recognizing such findings may improve the utilization of imaging modalities for patients with suspected urinary stone disease.
